# Metabolic regulations of a decoction of *Hedyotis diffusa* in acute liver injury of mouse models

**DOI:** 10.1186/s13020-017-0159-4

**Published:** 2017-12-20

**Authors:** Min Dai, Fenglin Wang, Zengcheng Zou, Gemin Xiao, Hongjie Chen, Hongzhi Yang

**Affiliations:** Traditional Chinese Medicine Department, The Third Affiliated Hospital of Sun Yat-sen University, Guangzhou, 510630 China

**Keywords:** *Hedyotis diffusa*, Metabolomics, Carbohydrate metabolism, Hepatitis, Mouse

## Abstract

**Background:**

Dysfunctional metabolisms are contributed to LPS/GALN-induced hepatitis. However, whether *Hedyotis diffusa* (HD) employs metabolic strategies against hepatitis is unknown.

**Methods:**

We use the cytokines expression, levels of serum alanine transaminase and aspartate transaminase, survival and histological analysis to measure the effect of decoction of HD on acute severe hepatitis of mouse induced by LPS/GALN. Meanwhile, we utilize GC/MS-based metabolomics to characterize the variation of metabolomes.

**Results:**

The present study shows the relieving liver damage in HD decoction-treated mice. Metabolic category using differential metabolites indicates the lower percentage of carbohydrates in LPS/GALN + HD group than LPS/GALN group, revealing the value of carbohydrate metabolism in HD decoction-administrated mouse liver. Further pathway enrichment analysis proposes that citrate cycle, galactose metabolism, and starch and sucrose metabolism are three important carbohydrate metabolisms that involve in the protective effect of decoction of HD during acute hepatitis. Furthermore, other important enrichment pathways are biosynthesis of unsaturated fatty acids, alanine, aspartate and glutamate metabolism, and arginine and proline metabolism. Fatty acids or amino acids involved in above-mentioned pathways are also detected in high loading distribution on IC01 and IC02, thereby manifesting the significance of these metabolites. Other key metabolites detect in ICA analysis were cholesterol, lactic acid and tryptophan.

**Conclusions:**

The variation tendency of above-mentioned metabolites is totally consistent with the protective nature of decoction of HD. These findings give a viewpoint that HD decoction-effected metabolic strategies are linked to underlying mechanisms of decoction of HD and highlight the importance of metabolic mechanisms against hepatitis.

**Electronic supplementary material:**

The online version of this article (10.1186/s13020-017-0159-4) contains supplementary material, which is available to authorized users.

## Background

The worldwide incidence of hepatocellular carcinoma (HCC), a major cause of human cancer death, has enhanced in recent years [1]. Hepatocyte death that drives liver disease progression from hepatitis associated with a number of liver insults, including steatosis, hepatotoxins, viral infection, and autoimmune disease, are responsible for the development of HCC [2, 3]. These liver insults are related to the subsequent development of inflammation, fibrosis, and cirrhosis. Actually, inflammation, a syndrome responsive to pathogen infection or injury, is a hallmark of liver disease that may represent a cause of HCC development [3, 4]. Therefore, it is an urgent clinical challenge to develop new therapeutic interventions through targeting hepatic inflammation, which may ultimately provide therapeutic benefit for the treatment of HCC [3].

*Hedyotis diffusa*, a traditional Chinese herbal medicine that belongs to the Rubiaceae family and also known as *Oldenlandia diffusa* and Bai Hua She She Cao [5], is widely spread in South of China and other Asian countries. *H. diffusa* has been largely employed in the treatments of inflammation-involved diseases, such as bronchitis, arthritis, rheumatism, appendicitis, sore throat, urethral infection, contusions, and ulcerations [6]. Accumulating evidence also proposes that *H. diffusa* is capable of controlling the liver, breast, lung, colon, brain and pancreatic cancers through promoting apoptosis of cancer cell and inhibiting tumor angiogenesis [7–11]. Moreover, the isolated splenocytes from *H. diffusa* extract-administrated leukemic mice manifest an improvement of T- and B- cell proliferation in vivo [12]. Also, *H. diffusa* addition affects the levels of cell markers (CD3, CD11b, and CD19) in white blood cell, enhances macrophage phagocytosis, and increases the cytotoxic activities of NK cells in normal Balb/c mice [13]. All above-mentioned studies show that *H. diffusa* has anti-inflammatory, anti-cancer and immunomodulatory activities. Actually, recent papers coincidentally demonstrate that inflammatory process, cancer development and progression, and immune response have strong inter-relationship with metabolism happened in host cells [14–17]. Currently, only one paper focuses on metabolic alterations of *H. diffusa* in tumor-bearing rat [18]. The underlying metabolic mechanisms related to *H. diffusa*-involved activities need to be elaborated further, and the metabolic activities of this herb in liver inflammation is unknown.

Metabolomics is a powerful new technology studying metabolic processes, identifying crucial biomarkers responsible for metabolic characteristics, and revealing metabolic mechanisms. Analysis of the key metabolites in various samples has become a meaningful part of improving the diagnosis, prognosis, and therapy of diseases [19]. Gas chromatography/mass spectrometry (GC–MS), liquid chromatography–mass spectrometry (LC–MS) and nuclear magnetic resonance (NMR) are three most common analytical technologies in metabolomics investigation [20]. While each technology has its own unique advantages and disadvantages, GC–MS is specifically becoming for the analyses of volatile compounds and thus is widely applied [21–23]. Here, we report the use of GC–MS combined with multivariate statistical tools to exploit, among the differential metabolites, key metabolites and important pathways as biomarkers capable of differentiating LPS/GALN treatment from the treatment of decoction of *H. diffusa* plus LPS/GALN in the liver metabolome.

## Methods

### Animals and experimental design

Adult female mice (C57BL/6, pathogen-free), weighing 24 ± 2 g from the same litters, were reared in an environmentally controlled breeding room (temperature: 20 ± 2 °C, humidity: 60 ± 5%, 12 h dark/light cycle), and in cages fed with sterile water and dry pellet diets. They were maintained in accordance with internationally accepted principles for laboratory animal use. All work was conducted in strict accordance with the recommendations in the Guide for the Care and Use of Laboratory Animals of the National Institutes of Health. The protocol was approved by the Institutional Animal Care (Animal Welfare Assurance Number: IS06). The Minimum Standards of Reporting Checklist (Additional file 1) contained details of the experimental design, and statistics, and resources used in this study.

According to the previous study [24], murine model of acute hepatitis was induced by combined injection of LPS (50 g/kg) and D-GALN (1.2 g/kg). Mice were randomly separated into 3 groups (*n* = 26 per group). The control and model groups were injected intraperitoneally with 200 μL of saline twice a day [6]; the treatment group received the 200 μL of decoction of *H. diffusa* (5 g/kg) twice a day [6]. Three days after injection, the mice in both model and treatment groups were challenged intraperitoneally by LPS/GALN. Twelve hours later, six mice in each group were euthanized by decapitation, and blood and livers were collected for following studies. The remaining 20 mice in each group were observed for 20 days to examine their survival.

### Preparation of a decoction of *H. diffusa*

According the previous procedure [5], 100 g of dried *H. diffusa* were cut into 1–1.5 mm pieces and crushed using a mortar and pestle, and then boiled with 1 L of deionised water for 1 h. After cooling, the decoction was centrifuged at 3000 rpm for 20 min, and filtered through a 0.45 μm filter. The filtrate was evaporated in vacuum (EYELA N-1001, Tokyo, Japan) and a dry residue was recovered. Finally, the total residue was reconstituted in saline and the final volume of extract is 100 mL (equal to 1.0 g raw material/mL).

### Histology analysis

The liver sample was fixed in 4% paraformaldehyde overnight at room temperature, then embedded in paraffin, sliced into 5-μm sections. For histological analysis, paraffin sections were stained with hematoxylin and eosin (H&E). The morphologic criteria used to determine the degree of necrosis included portal inflammation, hepatocellular necrosis, inflammatory cell infiltration, and loss of cell architecture. The pathological changes were evaluated in nonconsecutive, randomly chosen 200× histological fields.

### Quantitative RT-PCR assay

For quantitative RT-PCR assay, RNA was isolated from mouse livers using the TRIzol reagent according to the manufacturer’s instructions. 2 µg of total RNA was provided to generate the first-strand cDNAs by using commercially available kits (Applied Biosystems). All subsequent PCR reactions were carried out using the 7 Universal PCR Master Mix (Applied Biosystems). PCR primers of mouse CXCL1 were 5′-TCGTCTTTCATATTGTATGGTCAAC-3′ and 5′-CGAGACGAGACCAGGAGAAA C-3′. The primers for mouse TNFα were 5′-CATCTTCTAAAATTCGAGTGACAA-3′ and 5′-TGGGAGTAGACAAGGTACAACCC-3′. The real-time PCR primers for mouse IL-1β were 5′-ACAGATGAAGTGCTCCTTCCA-3′ and 5′-GTCGGAGATTCGTAGCTGGAT-3′. The real-time PCR primers for mouse MIP-2 were 5′-CCCCCTGGTTCAGAAAATCATC-3′ and 5′-AACTCTCAGACAGCGAGGCACATC-3′. The primers for the mouse housekeeping gene GAPDH were 5′-TTCACCACCATGGAGAAGGC-3′ and 5′-GGCATGGACTGTGGTCATGA-3′ and were used as a control.

### Measurement of cytokines in liver

Concentrations of TNF-α, IL-1β, IL-6, and MCP-1 in liver were measured through mouse-specific enzyme-linked immunosorbent assay (ELISA) kits (NeoBioscience, Shenzhen, China). Each analysis was carried out according to the manufacturer’s instruction, and the concentrations of cytokines were determined according to the standard curves.

### Measurement of serum alanine transaminase and aspartate transaminase activities

Blood samples were centrifuged at 1500*g* for 20 min at 4 °C, and alanine transaminase (ALT) and aspartate transaminase (AST) activities in serum were measured by commercial kits from Randox Laboratories (UK).

### Extraction of metabolites in mouse liver

For the metabolomics investigation, the extraction of total metabolites in mouse liver was performed according a procedure described previously [25]. Briefly, 1 g of liver tissue was homogenized and dissolved for 1 min in 1 mL of methanol at 4 °C. The homogenates were centrifuged at 12,000×*g* for 10 min at 4 °C. 300 μL of supernatant was transferred to a GC sampling vial containing ribitol (10 μL, 0.1 mg/mL), an internal standard, and then dried in a vacuum centrifuge concentrator before the subsequent derivatization.

### Derivatization and GC–MS analysis

Prior to GC–MS analysis, deriving liver samples was required. After drying samples, 80 μL of methoxamine/pyridine hydrochloride (20 mg/mL) was added to induce oximation for 1.5 h at 37 °C and then 80 μL of MSTFA, a derivatization reagent (Sigma), was mixed and reacted with the liver sample for additional 0.5 h at 37 °C. By centrifuging, 1 μL of supernatant derivative was added to a tube and analyzed using GC–MS (Trace DSQ II, Thermo Scientific). The separation conditions of GC–MS consisted of an initial temperature of 70 °C (5 min) with a uniform increase to 270 °C at a speed of 2 °C/min (5 min); 0.5 μL sample volume, splitless injection; injection temperature, 270 °C; interface temperature, 270 °C; ion source (EI) temperature, 30 °C; ionization voltage, 70 eV; quadrupole temperature, 150 °C; carrier gas, highly pure helium; velocity, 1.0 mL/min; and full scan way, 60–600 *m*/*z*.

### Statistical and bioinformatics analysis

The data of liver metabolome were collected using Thermo Foundation 1.0.1. The sum abundance value was employed for normalizing the resulting data matrix, and then the computed abundance of metabolites was centered for each tissue sample on their median value and scaled by their inter-quartile range (IQR) to decline between-sample variation [25, 26]. The significant analysis of microarray (SAM), a permutation-based hypothesis testing method for the analysis of proteomic and metabolomic data [27, 28], was applied to analyze the differential metabolites. Independent component analysis (ICA) was chosen as the pattern recognition method [29]. Statistical significance between groups was determined with the unpaired two-tailed Student *t* test. All data were analyzed by Prism (GraphPad Software, Inc.), and *P* values less than 0.05 and 0.01 were deemed as two significant levels.

## Results

### Decoction of *H. diffusa* (HD) attenuates the acute inflammation in hepatitis mouse

**Fig. 1:**
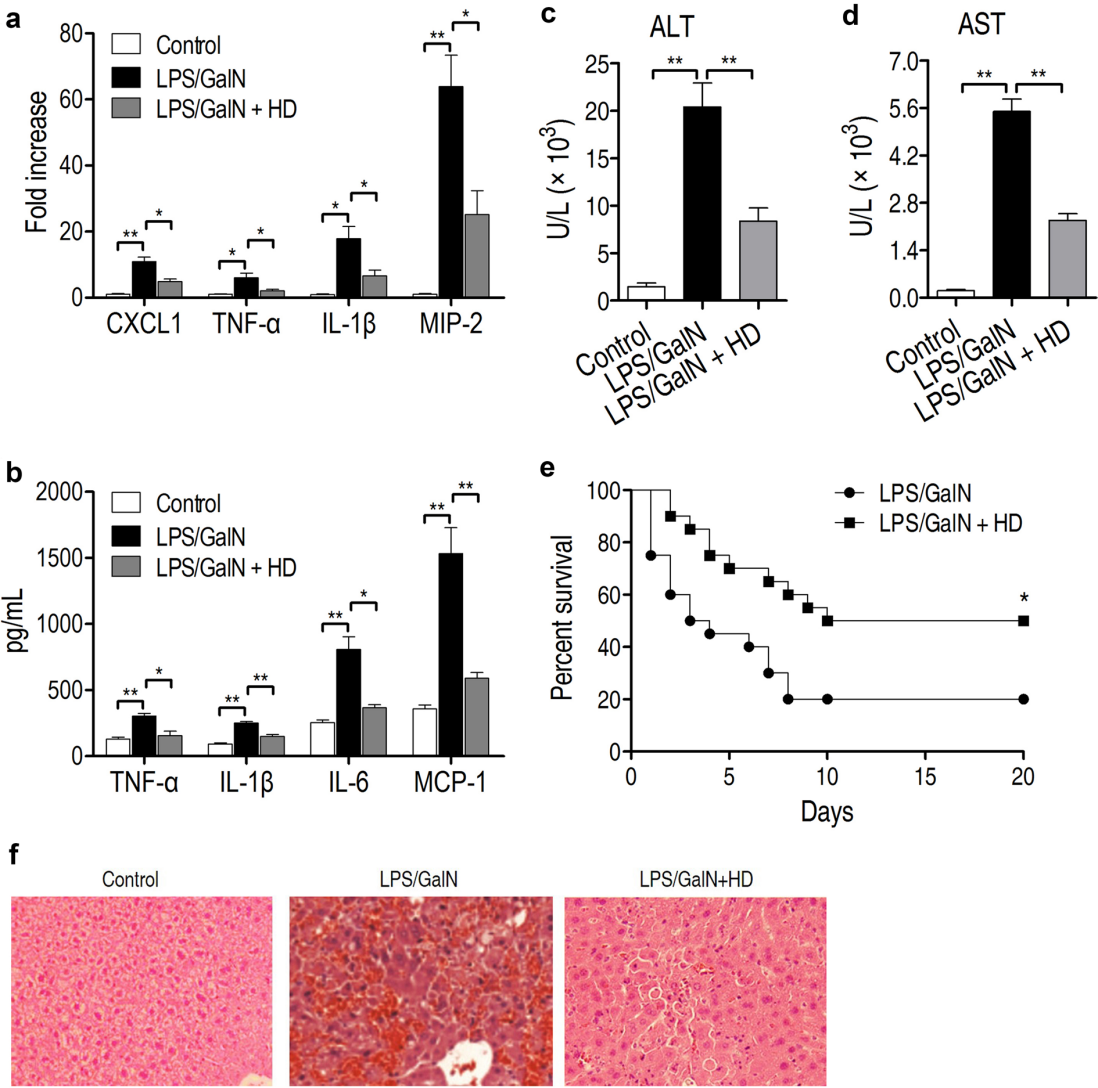
Decoction of *Hedyotis diffusa* (HD) relieves the acute inflammation in hepatitis mouse. **a** Administration of HD decoction blocked LPS/GALN-induced up-regulation of mRNA levels of CXCL1, TNF-α, IL-1β, MIP-2. **b** The treatment of HD decoction reduced the expressed levels of TNF-α, IL-1β, IL-6 and MCP-1 in liver of LPS/GALN-injected mice. **c** Alanine transaminase (ALT) level was measured in control or LPS/GALN- and LPS/GALN + HD-treated mouse. HD decoction significantly reduced the level of ALT (n = 5). **d** Aspartate transaminase (AST) level was measured in control or LPS/GALN- and LPS/GALN + HD-treated mouse. HD decoction significantly reduced the level of AST (n = 5). **e** Survival of hepatitis mouse was measured after treatment of HD decoction (n = 20, per group) within 20 days. **f** Effect of HD on LPS/GALN-mediated liver histopathologic changes

Normally, the most important criterion in evaluating a potential drug against hepatitis is its efficacy in vivo. Thus, we employed a hepatitis mouse model reported previously and revealed the potential effect of decoction of *H. diffusa* (HD) on acute inflammation. In brief, decoction of HD or saline control was injected into C57BL/6 mice 3 days and LPS/GALN was applied subsequently to induce the liver damage. Firstly, the mRNA levels of CXCL1, TNFα, IL-1β and MIP-2 gene, and productions of vital cytokines in liver were measured (Fig. 1a, b). Injection of LPS/GALN caused a significant elevation in the mRNA levels of CXCL1, TNFα, IL-1β and MIP-2 gene, and increased the secretions of TNFα, IL-1β, IL-6 and MCP-1 in mouse liver. The administration of decoction of HD (5 g/kg) had an ability to decrease the mRNA levels of these genes and production of these cytokines. Furthermore, the levels of alanine transaminase and aspartate transaminase were also reduced in serum of HD decoction-treated mice (Fig. 1c, d). By using survival analysis, we found that LPS/GALN led to rapid death of animals as early as 24 h after injection (Fig. 1e). Importantly, the application of decoction of HD delayed the incidence of death and increased the survival rate by twofolds, when compared with the saline control (Fig. 1e). The histological analysis of mouse liver in the control group indicated normal liver lobular architecture and cell structure (Fig. 1f). However, livers exposed to GalN/LPS presented numerous and extensive areas of portal inflammation and cellular necrosis and a significant increase in inflammatory cell infiltration. These changes were rescued by the application of decoction of HD (5 g/kg). Taken together, these data suggest that decoction of HD is an efficient Chinese Medicine that attenuates LPS/GALN-induced liver inflammation in vivo.

### Metabolomic profiling of mouse liver

**Fig. 2:**
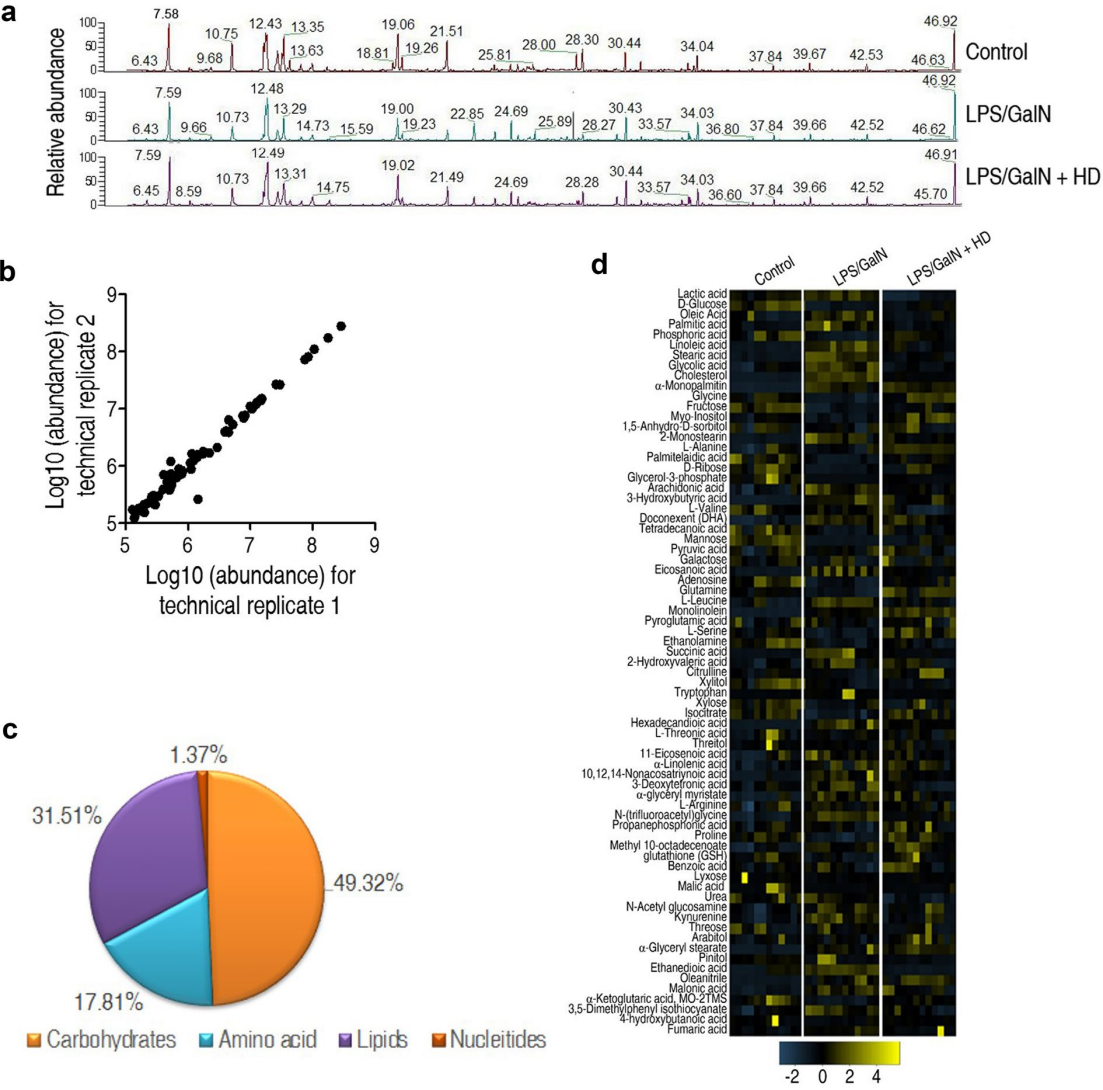
Metabolomic profiling of mouse liver. **a** Representative total ion current chromatograms from control, the LPS/GALN and LPS/GALN + HD samples. **b** Reproducibility of metabolomic profile platform used in the discovery phase. The abundances of metabolite quantified in cell samples over two technical replicates are presented. Correlation coefficient between technical replicates varies between 0.995 and 0.999. This plot reveals the two replicates with the smallest correlation of 0.995. **c** Metabolic category of recognized metabolites. **d** Heat map exhibiting the 73 metabolites. Yellow and steelblue indicate increase and decrease of metabolites relative to the median metabolite level, respectively (see color scale)

To identify the vital metabolic pathways and important metabolites that acted the helpful effect of decoction of HD on mouse hepatitis, GC–MS was employed to quantitatively evaluate the level of known metabolites in mouse livers which were obtained from six individuals each group. Typical total ion current chromatograms (TIC) were presented in Fig. 2a. 73 metabolites with dependable signal were found each sample. The correlation coefficient of two technical repeats revealed the reliability of the detection technology (Fig. 2b). The category showed that 49.32, 17.81, 31.51 and 1.37% of metabolites belonged to carbon sources, amino acids, lipids and nucleotides, respectively (Fig. 2c). Heat map showed the abundance of the metabolites, which came from the three groups (Fig. 2d). These data indicated that a carbohydrates-, amino acids- and lipids-dominant metabolome of mouse liver is developed. The metabolome varied among the three groups, suggesting an association between the metabolomics responses and degree of liver damage.

### Decoction of HD varied the metabolomic profiling of liver in LPS/GALN-injected mice

**Fig. 3:**
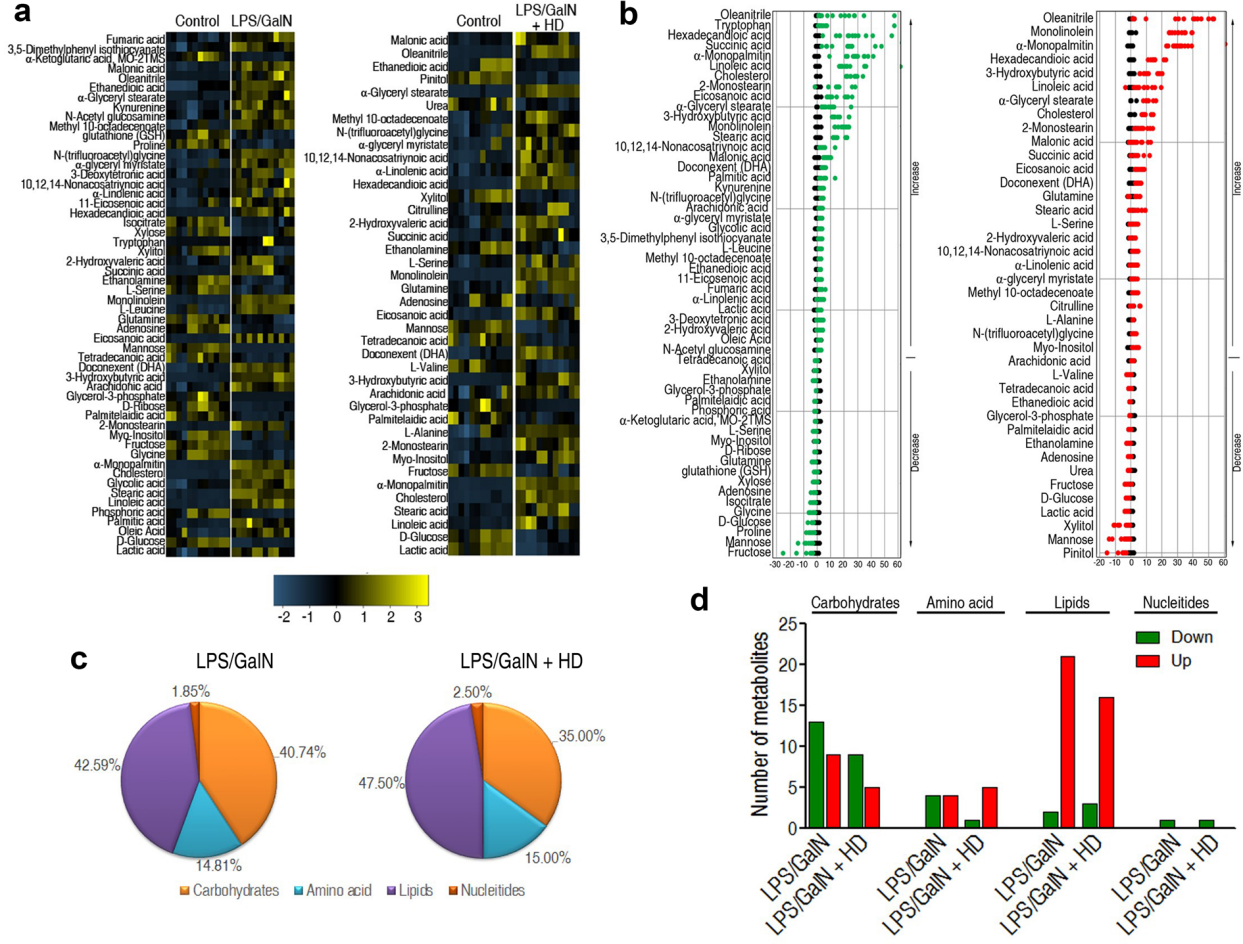
Varied metabolomes differentiating LPS/GALN + HD from LPS/GALN in mouse liver. **a** Heat map revealing relative abundance of 54 and 40 significantly varied metabolites in the LPS/GALN and LPS/GALN + HD as indicated, respectively. **b** Z-scores (standard deviation from average) corresponding to data in **a**. Left, the LPS/GALN group; right, the LPS/GALN + HD group. **c** Percentage of varied metabolites in four categories. **d** The number of metabolites increased and decreased in different categories

To further investigate a changed metabolome identifying the LPS/GALN + HD group from the LPS/GALN group, a two-sided Wilcoxon rank-sum test coupled with a permutation test was applied to detect differential metabolites. Fifty-four (73.97%) and forty (54.79%) metabolites out of the 73 metabolites were differential at *P* < 0.05 in LPS/GALN and LPS/GALN + HD group (Fig. 3a), respectively. Z value based on the control group was calculated for comparative study. Z score plot showed that it spanned from − 25.41 to 66.78 in LPS/GALN group and from − 15.61 to 55.21 in LPS/GALN + HD group (Fig. 3b). Higher varied abundances of metabolites were found in the LPS/GALN group than in the LPS/GALN + HD group. Notably, 20 metabolites down-regulated and 34 metabolites were up-regulated in the LPS/GALN group, while 14 were metabolites decreased and 26 metabolites increased in the LPS/GALN + HD group (Additional file 2).

Metabolic categories of these differential metabolites in abundance were explored further. They presented analogous varying percentage in the two groups, ranking lipids > carbohydrates > amino acids > nucleotides, but relative higher percentage of lipids and amino acid, and lower carbohydrates were discovered in the LPS/GALN + HD than the LPS/GALN groups (Fig. 3c). Figure 3d visualized the numbers of up-regulated and down-regulated metabolites in these categories. HD reduced the numbers of up-regulated and down-regulated carbohydrates caused by LPS/GALN, and up-regulated lipids and down-regulated amino acid in LPS/GALN group were declined after HD administration. These data reveal that decoction of HD might provide a helpful response through a change in metabolome.

### Differential enriched pathways responsible for the helpful response induced by HD

**Fig. 4:**
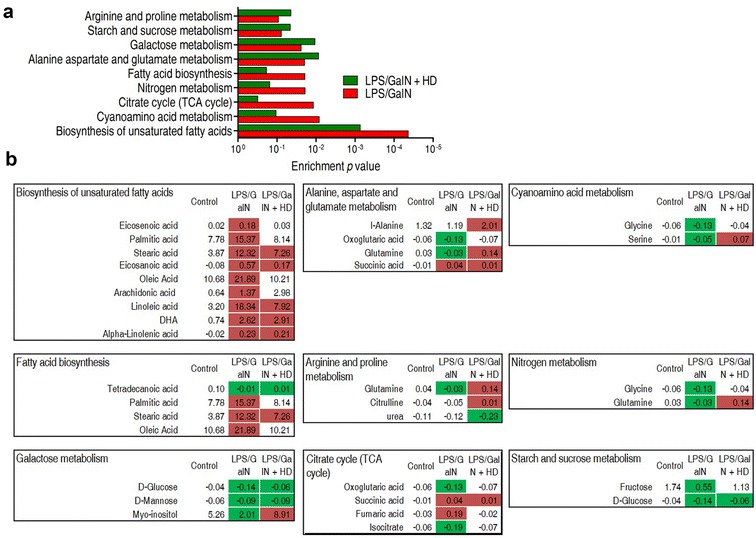
Pathway analysis and integrative analysis. **a** Pathway enrichment analysis of differential metabolites form LPS/GALN and LPS/GALN + HD using an online tool, Metaboanalyst 3.0 (http://www.metaboanalyst.ca/). Significantly enriched pathways are selected to plot. **b** Integrative analysis of metabolites in significantly enriched pathways. Up-regulation and down-regulation of metabolites are indicated as red and green, respectively. The number reveals the ratio of differential metabolites

To further investigate which pathways were enriched and what difference of the enriched pathways were detected between LPS/GALN and LPS/GALN + HD groups, an online tool, Metaboanalyst 3.0 was utilized. Shared and differential enriched pathways between them were showed in Fig. 4a. Three shared pathways were biosynthesis of unsaturated fatty acids, alanine, aspartate and glutamate metabolism, and galactose metabolism. Specifically, biosynthesis of unsaturated fatty acids was a pathway that had the lowest *P* value in both LPS/GALN and LPS/GALN + HD groups. Besides that, arginine and proline metabolism, and starch and sucrose metabolism were enriched only in LPS/GALN + HD group, and cyanoamino acid metabolism, citrate cycle and, nitrogen metabolism, and fatty acid biosynthesis were enriched only in LPS/GALN group.

Among these pathway, two were uniquely related to the relief of liver damage, which were arginine and proline metabolism, and starch and sucrose metabolism (Fig. 4b). Metabolites enriched in the starch and sucrose metabolism were all decreased. Although all metabolites enriched in biosynthesis of unsaturated fatty acids had higher abundance in LPS/GALN and LPS/GALN + HD groups in contrast to control group, abundance of most metabolites in LPS/GALN + HD group were lower than LPS/GALN group. In other words, HD was capable of reducing the abundance of these up-regulated metabolites enriched in biosynthesis of unsaturated fatty acids in LPS/GALN-treated liver. These metabolites included eicosenoic acid, palmitic acid, stearic acid, eicosanoic acid, oleic acid, arachidonic acid and linoleic acid. More importantly, some of metabolites in alanine, aspartate and glutamate metabolism, arginine and proline metabolism, and galactose metabolism were reversal between LPS/GALN and LPS/GALN + HD groups. These metabolites included glutamine and myo-inositol, and were all declined in LPS/GALN group and augmented in LPS/GALN + HD group. Besides, l-alanine enriched in alanine, aspartate and glutamate metabolism and citrulline enriched in arginine and proline metabolism was only boosted, and urea enriched in arginine and proline metabolism was only reduced in LPS/GALN + HD group. Moreover, three of four metabolites (oxoglutaric acid, fumaric acid and isocitrate), which were enriched in citrate cycle and showed higher abundance in LPS/GALN group, had the similar metabolic levels between control and LPS/GALN + HD groups. Meanwhile, remaining metabolite, succinic acid, had a lower abundance in LPS/GALN + HD group, when compared to the LPS/GALN group. Collectively, these results indicated that biosynthesis of unsaturated fatty acids, alanine, aspartate and glutamate metabolism, arginine and proline metabolism and citrate cycle might be significantly related to the HD decoction-induced benefit for hepatitis mouse.

### Identification of crucial metabolites using ICA analysis

**Fig. 5:**
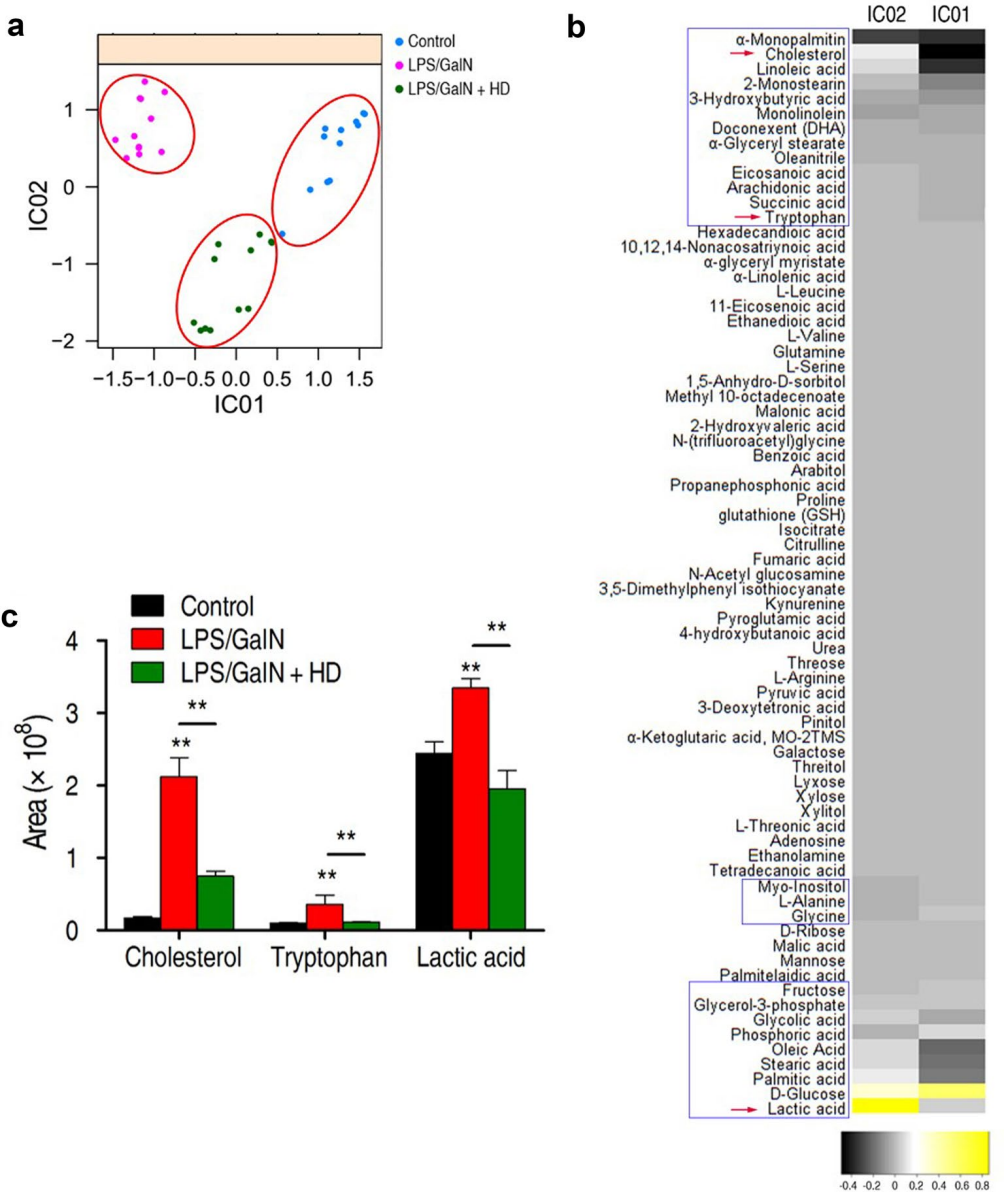
Investigation of vital metabolites that separate LPS/GALN + HD from the LPS/GALN using independent component analysis (ICA). **a** ICA directly represents variation of metabolites among control, LPS/GALN and LPS/GALN + HD. Each dot in the plot represents the replicate analysis of samples. **b** The weight distribution on IC01 and IC02 for the metabolites are shown. The weight (also called loadings) are proportional to the importance or significance of a metabolite for a corresponding independent component, in other words, the observed biological phenomenon. The interpretations of IC01 and IC02 correspond to **a**. Blue box indicates the metabolites which have largest the loadings. **c** Comparison of cholesterol, tryptophan, and lactic acid among control, LPS/GALN and LPS/GALN + HD. Error bars ± SD, **P < 0.01

ICA was an available and alternative application to be designed to recognize the sample pattern. As shown in Fig. 5a, control group and LPS/GALN group was separated obviously on IC01, and IC02 depicted the obvious differentiation between LPS/GALN and LPS/GALN + HD groups. Because of a clear sample discrimination by ICA in Fig. 5a, it was possible to detect the significant metabolites differentiating LPS/GALN and LPS/GALN + HD. In Fig. 5b, the loadings of different independent component IC01 and IC02 were visualized in a heat map. Ranking of the varied metabolites displayed that 25 metabolites (blue boxes in Fig. 5b) have the largest loading in IC01 and IC02. Out of these metabolites, d-glucose, fructose, palmitic acid, stearic acid, oleic acid, glycine, l-alanine, myo-inositol, succinic acid, arachidonic acid, eicosanoic acid, DHA, 3-hydroxybutyric acid and linoleic acid were the shared significant metabolites found in above pathway enrichment analysis, while others were the new crucial metabolites detected in ICA analysis. More interestingly, only tryptophan, lactic acid and cholesterol were differentiated significantly between control and LPS/GALN group, as well as between LPS/GALN and LPS/GALN + HD group (Fig. 5c).

## Discussion

**Fig. 6:**
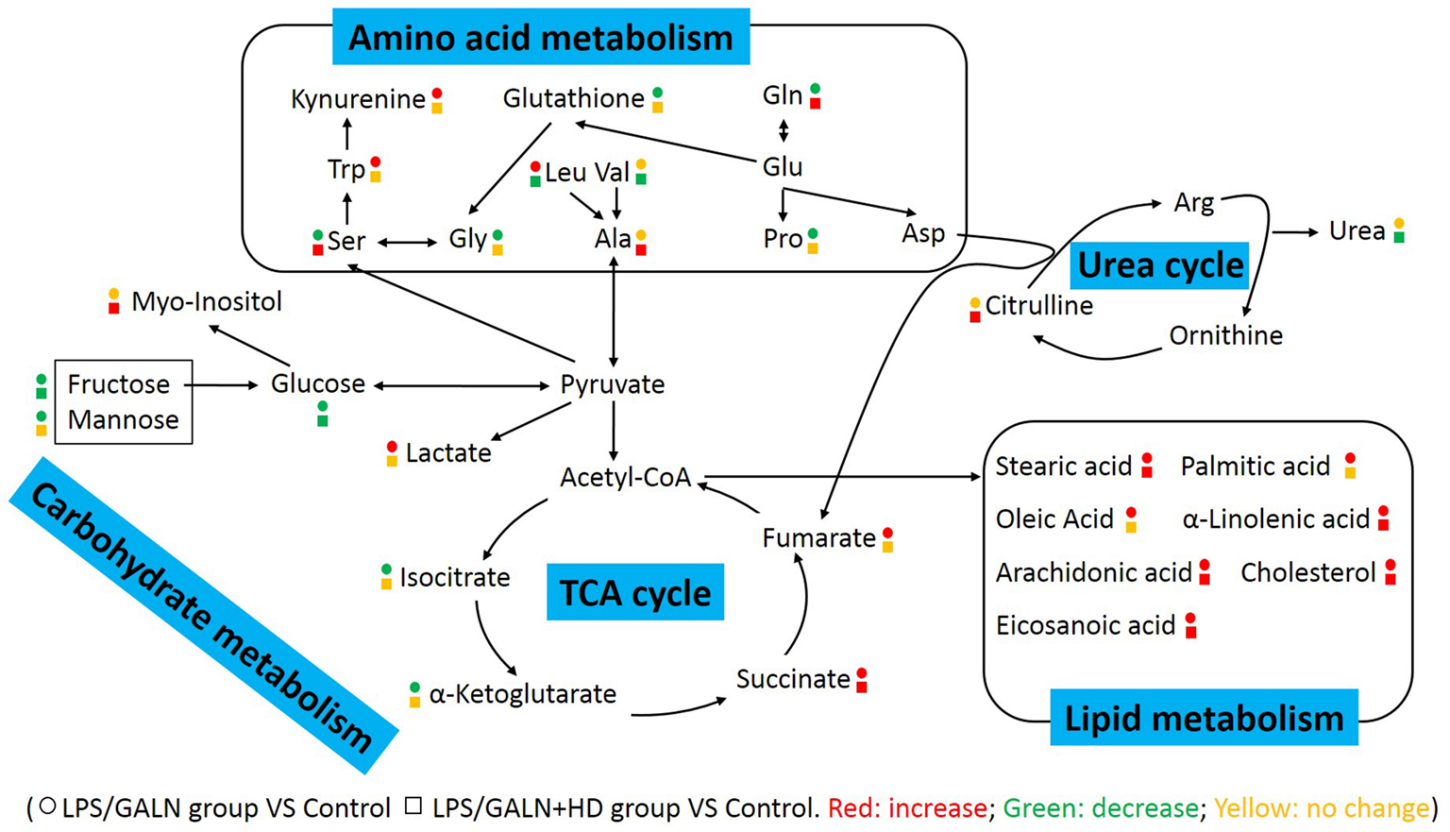
Potential metabolic mechanisms of LPS/GALN-induced liver injury and protection by *H. diffusa* (HD). Symbol circle and square represent the relative metabolite changes in the LPS/GALN-injected group and the HD treatment group, respectively. The decrease, increase and no change in levels with statistical significance are presented in green, red and yellow, respectively

The latest new in anticancer study suggests that *H. diffusa* treatment is capable of reducing the injury caused by Walker 256 tumor and maintaining a metabolic balance [18]. In addition, recent evidence reveals that the dysfunctional metabolisms are responsible for LPS/D/GALN-induced acute hepatitis [30, 31]. However, it is unknown that whether HD may protect host against LPS/D/GALN-induced liver damage through mounting metabolic strategy. Therefore, in the present study, we focus on the examination of metabolic response of HD decoction-pretreated acute hepatitis through using GC/MS-based metabolomic. Our study not only reveals that metabolic response is likely linked to the degree of LPS/D/GALN-induced acute hepatitis through overall understanding the metabolomes among control, LPS/D/GALN and LPS/D/GALN + HD groups, but also detects some crucial pathways and key metabolites (Fig. 6).

The finding of current metabolic category shows that LPS/D/GALN + HD have a lower percentage of carbohydrates than LPS/GALN group (Fig. 3c, d), subsequent pathway enrichment analysis further make clear that citrate cycle, galactose metabolism, and starch and sucrose metabolism are able to be involved in the metabolic activities of HD. Among these, citrate cycle is more striking because two metabolites (oxoglutaric acid and isocitrate) are down-regulated but others (succinic acid and fumaric acid) are up-regulated after LPS/GALN treatment (Fig. 6). Consistent with this, previous study show the down-regulation of isocitrate, and up-regulation of succinic acid and fumaric acid in LPS-treated mice [32]. In fact, recent papers demonstrate that LPS stimulates a profound metabolic transition to aerobic glycolysis through phosphatidyl inositol 3′-kinase/Akt pathway and inhibits mitochondrial oxidative phosphorylation, an action that have an indispensable connection with citrate cycle [33, 34]. Succinic acid is an inflammatory signal that activate IL-1β through stabilizing the hypoxia-inducible factor-1α (HIF-1α) [32]. Similarly, fumaric acid also have a function in HIF stability and fumaric acid up-regulation can be recognized as a tumor-promoting event [35]. Given that the lower abundance of succinic acid and fumaric acid are found in LPS/D/GALN + HD group than that in LPS/D/GALN group, thus a becoming possibility is that modulation of succinic acid and fumaric acid concentrations and following HIF-dependent cytokine production partly explains how decoction of HD possesses strong host protection in liver damage. Other interesting carbohydrates are d-glucose and myo-inositol enriched by pathway analysis (Fig. 4b), and lactic acid detected by ICA analysis (Fig. 5c). In patients, glucose metabolism is abnormal when the liver cell damage occurs [36], and the higher level of lactic acid in LPS/GALN group can be explained by the acceleration of aerobic glycolysis [33, 34]. After HD decoction treatment, the abundance of d-glucose and lactic acid is almost equal to the control group, indicating that maintaining the normal glucose metabolism and glycolysis is the potential metabolic mechanisms to relieve the acute hepatitis. It has been reported that myo-inositol plays an important role in immunity system [25, 37]. However, the detailed meaning of myo-inositol elevated by decoction of HD still needs to be studied in future.

Combining current pathway enrichment analysis with ICA analysis, the shared lipid-related metabolites are palmitic acid (PA), stearic acid (SA), oleic acid (OA), linoleic acid (LA), eicosanoic acid (EA) and arachidonic acid (AA). We know that PA (16:0), stearic acid (18:0), OA (18:1) and LA (18:2) are capable of stimulating TLR4 signaling to produce an inflammatory response [38], which may eventually contribute to acute severe hepatitis [39]. Other lipid-related metabolites are AA and cholesterol, which also have an excellent action in inducing the severe inflammation [40]. These metabolites were up-regulated by LPS/GALN treatment but decoction of HD down-regulated these metabolites, indicating that decoction of HD has an anti-inflammatory action through declining the abundance of lipids including PA, SA, OA, LA, AA and cholesterol (Fig. 6).

For amino acid metabolisms, alanine, aspartate and glutamate metabolism, and arginine and proline metabolism, cyanoamino acid metabolism, and nitrogen metabolism are enriched by pathway analysis. In these pathway, l-alanine, l-glutamine, citrulline, glycine and serine are involved (Fig. 6). l-Alanine, a significant energy substrate for cell, is beneficial for supporting gluconeogensis and leucocyte metabolism through unknown mechanism [41]. l-Glutamine is known to support the anti-inflammatory response through various signal pathways [42–44]. Serine and glycine are two potent antioxidants that scavenge free radicals, thereby playing an essential role in anti-oxidative defense of liver cell [45, 46]. There is also in vivo evidence that glycine blunts the production of TNFα and reduces inflammatory reactions [47, 48]. When compared to the LPS/GALN group, the boosted level of l-alanine, l-glutamine, glycine and serine found in LPS/GALN + HD group indicates that the high level of these amino acids is benefit for the alleviation of acute severe hepatitis. Citrulline and nitric oxide (NO) are produced by the metabolic response of l-arginine through NO synthase (NOS) and l-arginine also can generate urea and ornithine through arginase [23, 49]. The boosted citrulline and declined urea in LPS/GALN + HD revealed that NO may be involved in the protective effect of HD decoction on acute severe hepatitis, which certainly requires to be determined in further investigation. In addition, tryptophan is a high loading in differentiating LPS/GALN + HD from LPS/GALN. Tryptophan metabolism is increased during inflammation or stimulation by LPS or certain cytokines [50]. Most of tryptophan in mammals is oxidized along kynurenine pathway and kynurenine promotes carcinogenesis by acting on the aryl hydrocarbon receptor [51]. Thus inhibiting tryptophan metabolism is a possible metabolic mechanisms for HD decoction-induced protection.

## Conclusions

The current study uses GC/MS-based metabolomics to characterize variation of metabolomes in response to LPS/GALN and HD decoction treatment before LPS/GALN. Metabolic category using differential metabolites showed the lower percentage of carbohydrate in LPS/GALN + HD group than LPS/GALN group, revealing that carbohydrates metabolism may play an important role in HD-treated mice to combat liver damage. Subsequent pathway enrichment analysis further find out that citrate cycle, galactose metabolism, and starch and sucrose metabolism are three important carbohydrate metabolisms that involve in the protective effect of decoction of HD during acute severe hepatitis. Thus, these findings provide a viewpoint that underlying mechanisms of decoction of HD are connected to the metabolic strategies and highlight the value of metabolic strategies against hepatitis.

## Additional files

**Additional file 1.** The minimum standards checklist.

**Additional file 2.** The up-regulated and down-regulated metabolites in different categories among control, LPS/GALN group and LPS/GALN + HD group.
